# A Low Voltage Liquid Crystal Phase Grating with Switchable Diffraction Angles

**DOI:** 10.1038/srep39923

**Published:** 2017-01-05

**Authors:** Haiwei Chen, Guanjun Tan, Yuge Huang, Yishi Weng, Tae-Hoon Choi, Tae-Hoon Yoon, Shin-Tson Wu

**Affiliations:** 1 College of Optics and Photonics, University of Central Florida, Orlando, Florida 32816, USA; 2 School of Electronic Science and Engineering, Southeast University, Nanjing 210018, China; 3 Department of Electronics Engineering, Pusan National University, Busan 46241, Korea

## Abstract

We demonstrate a simple yet high performance phase grating with switchable diffraction angles using a fringe field switching (FFS) liquid crystal (LC) cell. The LC rubbing angle is parallel to the FFS electrodes (i.e. α = 0°), leading to symmetric LC director distribution in a voltage-on state. Such a grating exhibits three unique features: 1) Two grating periods can be formed by controlling the applied voltage, resulting in switchable diffraction angles. In our design, the 1^st^ diffraction order occurs at 4.3°, while the 2^nd^ order appears at 8.6°. 2) The required voltage to achieve peak diffraction efficiency (*η*~32%) for the 1^st^ order is only 4.4 V at λ = 633 nm as compared to 70 V for a conventional FFS-based phase grating in which α ≈ 7°, while the 2^nd^ order (*η*~27%) is 15 V. 3). The measured rise and decay time for the 1^st^ order is 7.62 ms and 6.75 ms, and for the 2^nd^ order is 0.75 ms and 3.87 ms, respectively. To understand the physical mechanisms, we also perform device simulations. Good agreement between experiment and simulation is obtained.

Liquid crystal (LC) phase grating has found useful applications in laser beam steering^1^, fiber-optic communications^2^, programmable shaping of femtosecond pulses^3^, and eye-tracking for the emerging virtual reality displays^4^,^5^. For a nematic LC-based phase grating, its operation voltage is usually lower than 15 V, depending on the dielectric anisotropy (Δ*ε*) of the employed LC mixture^6^,^7^, however, slow response time (typically > 10 ms)^8^,^9^ is a major concern. To achieve sub-millisecond response time and 2π phase change, polymer-stabilized blue phase liquid crystal (BPLC)^10^,^11^,^12^ and polymer network liquid crystal (PNLC)^13^ have been developed. However, these transmission-type phase gratings suffer from high operation voltage and noticeable hysteresis. Especially, hysteresis affects the accurate beam control and should be eliminated.

Recently, a dual-period tunable phase grating using BPLC has been proposed^14^,^15^. In addition to tunable diffraction efficiency, the diffraction angle can also be switched by the applied voltage. Thus, it offers more options for applications, such as optical interconnects. Nevertheless, the driving scheme is quite sophisticated and the operation voltage of the BPLC phase grating is still as high as 100 V. Moreover, the ion accumulation approach^15^ using a biased voltage may lead to long term instability. Therefore, there is urgent need to develop a simple grating structure yet keeping low voltage, fast response time, and switchable diffraction angle.

In this paper, we propose a new yet simple phase grating with switchable diffraction angles using a fringe-field-switching (FFS) LC cell. The key part of our design is that the LC rubbing direction is parallel to the interdigitated electrodes, i.e. α = 0°. By controlling the applied voltage, the diffraction angle can be switched between the 1^st^ order and the 2^nd^ order in less than 10 ms. Meanwhile, the peak diffraction efficiency reaches 32.2% for the 1^st^ order, which is ~80% of the theoretical limit (~41%), and 27.5% for the 2^nd^ order, while the contrast ratio exceeds 1000:1. To understand the physical mechanism, we also performed device simulation. Good agreement between experiment and simulation is achieved.

## Principle

**Figure 1:**
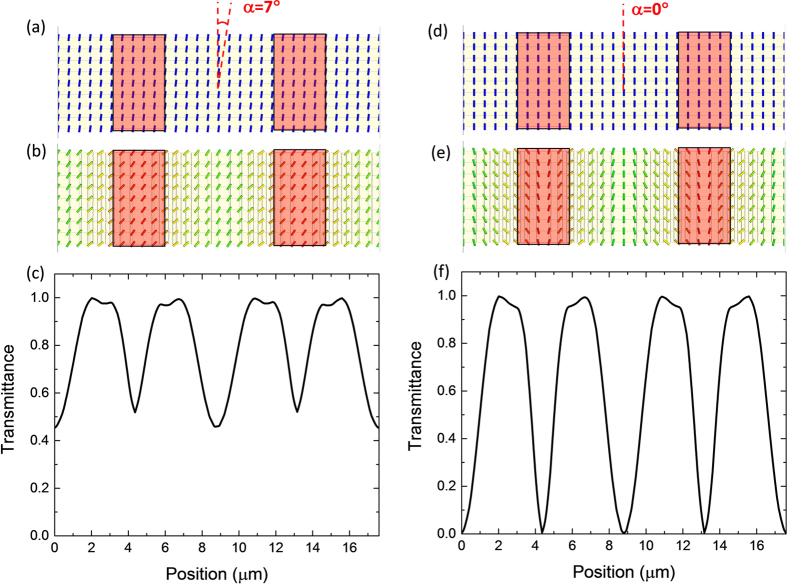
Top view for a FFS cell with (**a–c**) rubbing angle α = 7°, and (**d–f**) α = 0°. (**a**) and (**d**) LC director distribution at 0 V; (**b**) and (**e**) LC director distribution at *V*_*on*_; (**c**) and (**f**) position-dependent transmittance profile at *V*_*on*_. Red rectangles represent patterned pixel electrodes in the FFS cell; here LC with Δε > 0 is employed.

In FFS displays using a positive Δ*ε* LC, the rubbing angle is usually set at α = 7°~12° with respect to the interdigitated electrodes [Fig. 1(a)] so that the LC directors can rotate in one direction under the action of electric field [Fig. 1(b)]. Although the transmittance is not uniform across the electrodes, the average transmittance (this is what the eye perceives) is higher than 85% [Fig. 1(c)]. This is good for display^16^; however, it’s unacceptable for grating applications, because the refractive index variation is too small along the horizontal direction. To generate sufficient phase difference, a strong electric field is required. This explains why the operation voltage of a conventional FFS LC phase grating^17^ is as high as 70 V.

Recently, an FFS with zero rubbing angle (α = 0°) [Fig. 1(d)] was proposed to improve the LCD’s response time^18^. The basic concept is that LC directors are reoriented toward opposite directions at the edges of electrode, while in the middle of ITO electrodes and electrode gaps the LC directors remain stationary because the electric fields are symmetric but in opposite directions [Fig. 1(e)]. These standing regions make two important impacts. On the positive side, they confine the neighboring molecules, which helps shorten the decay time once the electric field is removed. However, in these areas there is no (or every little) phase retardation so that the transmittance is very low [Fig. 1(f)]. This is the major tradeoff for display applications, i.e. faster response time vs. lower transmittance. But for phase grating, it becomes an outstanding advantage because it overcomes the biggest technical hurdle on small refractive index change by providing sufficient phase difference at a relatively low voltage. Meanwhile, due to the symmetric LC distribution the grating constant is only half of the electrode period. As a result, a large diffraction angle, e.g. 2^nd^ order, can be achieved. For a conventional in-plane switching (IPS) based grating^10^,^19^, where the grating constant is determined by the electrode dimension, only the 1^st^ order is formed, thus the diffraction angle is quite limited (~3°). To enlarge diffraction angle, we can decrease the electrode dimension, but the device fabrication would be more challenging.

## Results and Discussions

**Figure 2:**
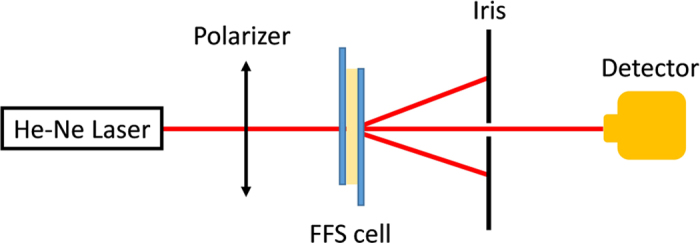
Experimental setup for characterizing the performance of our phase grating.

In experiment, we prepared an FFS cell with electrode width *w* = 2.8 μm, electrode gap *g* = 6 μm, and cell gap *d* = 3.4 μm. The rubbing direction was parallel to the patterned electrodes (*α* = 0°), while the pretilt angle (*θ*_*p*_) was controlled at 2°. An LC mixture with Δ*ε* = 5.1 was injected into the cell via capillary flow. Figure 2 shows the experimental setup for characterizing the phase grating. A He-Ne laser (*λ* = 633 nm) was used as probing beam. The transmission axis of the linear polarizer was set perpendicular to the pixel electrodes of the FFS cell. An iris was placed behind the FFS cell to select the diffraction order. The intensity of each order was detected by a photodiode in the far-field located at a distance of ~30 cm.

**Figure 3:**
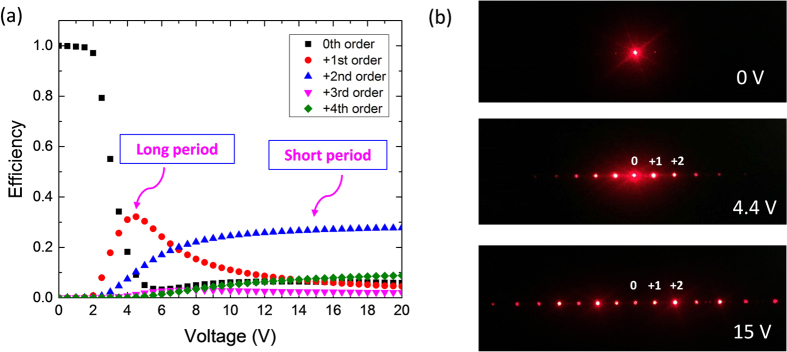
(**a**) Measured voltage-dependent diffraction efficiency for each diffraction order; (**b**) Diffraction pattern at different applied voltages: 0 V, 4.4 V and 15 V.

Figure 3(a) shows the measured diffraction efficiency for each order as the applied voltage increases. As expected, the operation voltage is quite low (<15 V), which means a sufficient phase difference is achieved, leading to strong diffraction effect. In contrast, the required voltage of a conventional FFS-based phase grating^17^ is ~70 V. In addition, another interesting feature is obtained unexpectedly. As Fig. 3(a) depicts, the diffraction efficiency (*η*) of the 1^st^ order depends on the applied voltage. Below 2V, the zeroth order dominates while the higher orders are hardly observed. As *V* > 2 V_rms_, *η* increases first, reaches ~32.2% (which is ~80% of the theoretical limit) at 4.4 V_rms_, and then gradually decreases. The contrast ratio of the 1^st^ order at *V* = 4.4 V_rms_ and *V* = 0 exceeds 1000:1. On the other hand, *η* of the 2^nd^ order increases gradually and then saturates as *V* > 10 V_rms_. That means the grating constant can be switched from a coarser one (i.e. period of FFS electrode) to a finer one (i.e. half period of the FFS electrode).

This unexpected property can be visualized clearly in Fig. 3(b). At the voltage-off state (*V* = 0), the diffraction effect results from the periodicity of electrodes is quite weak and the laser power is mainly on the 0^th^ order. As the applied voltage increases to 4.4 V, the ±1^st^ orders dominate. In this case, the grating constant is large, which is determined by the dimension of FFS electrode. If we further increase the voltage to 15 V, the 2^nd^ order becomes the dominant one, which corresponds to a smaller grating constant (i.e. half period of FFS electrode). This dual-period phase grating is much simpler than the previously reported approaches using BPLC^14^,^15^, and its driving voltage is also much lower.

For conventional diffraction angle control, each diffractive order would be expected to move with varying the diffractive angles. However, the switchable diffraction angles reported here is slightly different from the commonly considered switchable property. In our configuration, the grating constant is reduced by 2X, from a coarser one (i.e. period of FFS electrode) to a finer one (i.e. half period of the FFS electrode), so that resultant diffraction pattern is perfectly overlapped, as shown in Fig. 3(b). It is difficult to distinguish the movement of each diffractive order. But we can see clearly that the diffraction energy is transferred from odd orders (e.g. 1st order, 3rd order) to even orders (e.g. 2nd order, 4th order).

Next, we investigate each grating period in more details. For the long-period grating, its 1^st^ order diffraction efficiency peaks at 32.2% and the diffraction angle is 4.3°. This efficiency is twice higher than that of other nematic LC-based phase grating using IPS mode (*η*~16%)^6^, but somewhat lower than BPLC-based one (~40%)^11^. Finer phase profile helps improve the diffraction efficiency. BPLC exhibits rectangular-like spatial phase profile^11^, as a result, its peak diffraction efficiency is approaching the theoretical limit. Another important parameter for a tunable phase grating is response time. As discussed above, the stationary LC regions in our FFS cell help confine the neighboring molecules and accelerate the transition process. Indeed, the measured rise time is 7.62 ms and decay time is 6.75 ms when switching between 0 and 4.4 V, which is about 2X faster than the conventional one (~15 ms)^18^.

For the short-period phase grating (Fig. 3(a)), the diffraction angle is doubled (8.6°) and its diffraction efficiency can be tuned from 0% to 27.5% (~70% of the theoretical limit) at 15 V. Such an operation voltage is ~7X smaller than that of a BPLC-based phase grating^10^,^14^,^15^. Again, due to the strong confinement effect from the standing layers our measured rise time is 0.75 ms and decay time is 3.87 ms. If an ultra-low viscosity LC mixture is employed, the response time can be further improved^20^.

To understand the underlying physical mechanism of this unexpected dual-period phase grating, we performed rigorous simulations using finite element method^21^ and 2 × 2 extended Jones matrix method^22^. Please note that for those LC gratings using uniform longitudinal electric field or blue phase liquid crystal, the polarization of incident light would not be changed. As a result, it would be relatively easy to calculate the phase profile by simple integrations along light propagation direction (i.e. z-axis). However, in our nematic FFS phase grating, the electric field is strong and nonuniform in both lateral and longitudinal directions, therefore, the polarization of incident light would be changed due to the rotation of LC molecules. This spatially varying polarization distribution effect is unique and critical, thus, we have to use the Jones matrix to track the polarization change.

Firstly, we compute the LC director distribution using the finite element method, and this step can be conducted with commercial software TechWiz LCD (Sanayi, Korea). Then we divide the LC medium into 40 layers to utilize Jones matrix methods to calculate the output wavefront of the light. Since the grating constant Λ ≫ λ, the light interference inside the grating is negligible and the periodic boundary condition can be applied here^23^. The direction parallel to the interdigitated electrode is defined as x-axis, and the direction perpendicular to the electrode is set as y-axis. In our experiment, the incident light is controlled to y-direction, which could be represented as ***J***_**0**_ = (0, 1).

Each LC layer could be considered as a wave plate and its corresponding Jones matrix is:


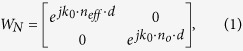


where *d* is the thickness of LC layer and *n*_*eff*_ is the effective index at the *N*^*th*^ layer:


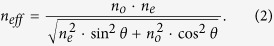


here, *θ* denotes the tilt angle of the LC directors. Therefore, the output electric field distribution along *y*-axis is the product of the Jones matrices of the whole LC layers and ***J***_**0**_^24^:


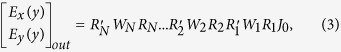


where *R*_*N*_ is the rotation matrix of the *N*^*th*^ layer and can be represented by:


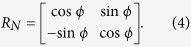


here, *φ* is defined as the azimuthal angle between the polarization of incident light and *x*-axis.

Since the diffracted light is detected by the photodiode at far field, here we can use the Fraunhofer diffraction equation to model the diffraction pattern^25^. Based on the calculated wavefront of output light, fast Fourier transform is conducted:


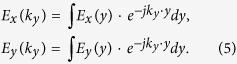


Then the output intensity can be calculated from following equation:


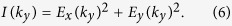


**Figure 4:**
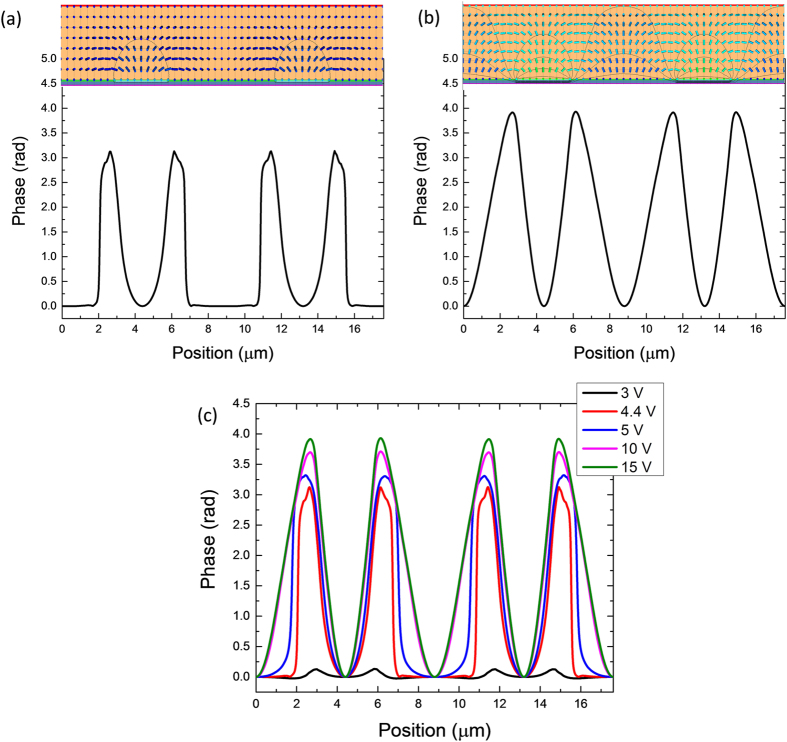
Simulated LC director distribution and calculated phase profile of the output light at (**a**) 4.4 V and (**b**) 15 V; (**c**) Calculated phase profile of the output light at different applied voltages.

Figures 4(a) and 4(b) show the simulated LC director distribution (upper part) at 4.4 V and 15 V, respectively, and the resultant phase profile of the output light (lower part). In the low voltage region, the LC director reorientations mainly occur near the electrode edges, whereas in the middle of electrode gap most of the LC directors do not move. This is clearly indicated by the phase profile of the output light (Fig. 4(a)). The phase is almost unchanged in the middle of electrode gap (flat region), but large phase accumulation arises near the electrode edges. Therefore, the large grating constant (dimension of FFS electrode) dominates, although there are some variations within each period. As the voltage increases to 15 V, most of the LC directors are reoriented by the strong electric field [Fig. 4(b)]. In this case, small grating constant starts to form and eventually dominates. Clear pattern with half period of FFS electrode is illustrated in the phase profile in Fig. 4(b). Figure 4(c) shows the continuous transition from large grating constant to small one as the operation voltage increases gradually. Also from Fig. 4(c), as the voltage increases from 10 V to 15 V, the phase profile only increases slightly, which means the diffraction efficiency saturates in the high voltage region, as shown in Fig. 3(a).

**Figure 5:**
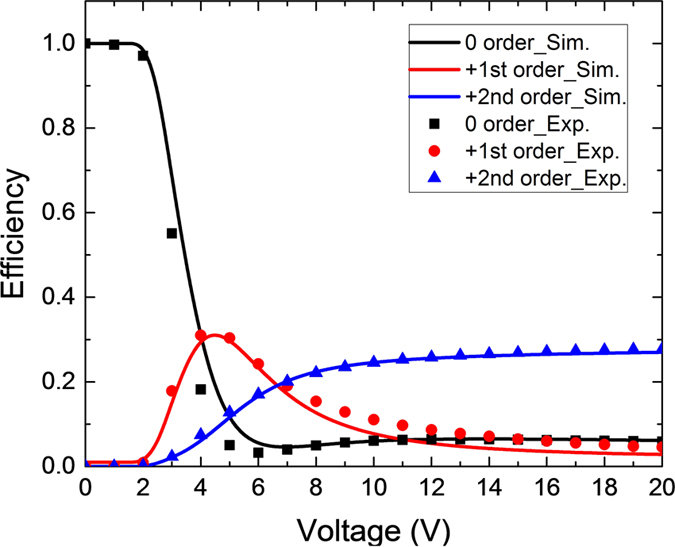
Calculated voltage-dependent diffraction efficiency curve for each order (Dots: measured data, solid curves: simulation results). Please note positive and negative orders have the same diffraction efficiency.

To validate the above simulation results, we also conducted experiment. Results are plotted in Fig. 5, where solid lines represent the simulation results and dots stand for the experimental data. From Fig. 5, the dual-period property is accurately predicted and diffraction efficiencies agree well with experiment. Small discrepancy in the high voltage region for the 1^st^ order may originate from the fabrication errors, including the variations of electrode structure, cell gap or passivation layer sandwiched between pixel and common electrodes.

**Figure 6:**
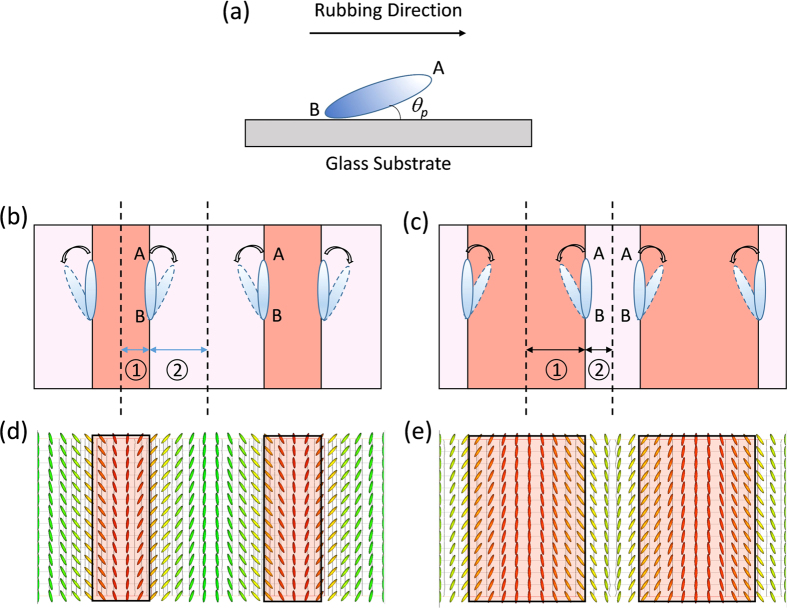
(**a**) Pretilt angle induced by the rubbing of alignment layer on a glass substrate; (**b**) and (**d**) Schematic diagram and simulated LC director distribution of an FFS cell with electrode width *w* = 2.8 μm and electrode gap *g* = 6 μm; (**c**) and (**e**) are with electrode width *w* = 6 μm and electrode gap *g* = 2.8 μm. The darker red regions represent pixel electrodes.

Another potential concern for our new FFS cell with α = 0° is the disclination line, because the LC directors do not know which direction to reorient (i.e. clockwise or counterclockwise) under the perpendicular electric field. However, this uncertainty exists only when we consider a small isolated local area. In fact, such FFS cell should be analyzed as an indivisible unit. Therefore, we use Gibbs free energy to analyze this phenomenon^26^:


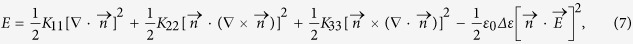


where *K*_*11*_, *K*_*22*_, and *K*_*33*_ are the splay, twist, and bend elastic constants and 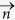 is the unit vector of LC director. In Eq. (7), the free energy contributed from flexoelectric effect is not considered. When an external electric field is present, the LC directors have to overcome the elastic force and anchoring force in order to reorient. For our zero-rubbing FFS cell, the electrode width and gap are not identical, e.g. electrode width *w* = 2.8 μm and electrode gap *g* = 6 μm, as Fig. 6(b) depicts. Let us consider the region between dashed lines, which is one half period of the whole structure. Clearly, region 1 is much smaller than region 2. Therefore, to obtain the lowest Gibbs free energy of the entire system, LC directors tend to rotate clockwise, as the arrow indicates. If the LC directors rotate counterclockwise, the strong squeezing effect within a small area (region 1) would undoubtedly increase the total Gibbs free energy. This has been proven by the simulation results [Fig. 6(d)] using TechWiz LCD, where director distribution is calculated by solving the total Gibbs free energy [Eq. (7)]. To further verify our hypothesis, we investigate another zero-rubbing FFS cell with different electrode configuration: electrode width *w* = 6 μm and electrode gap *g* = 2.8 μm [Fig. 6(c)]. In this case, region 1 is larger than region 2, so that LC directors tend to rotate counterclockwise. This is also validated by the simulated LC director distribution [Fig. 6(e)] by solving Eq. (7).

**Figure 7:**
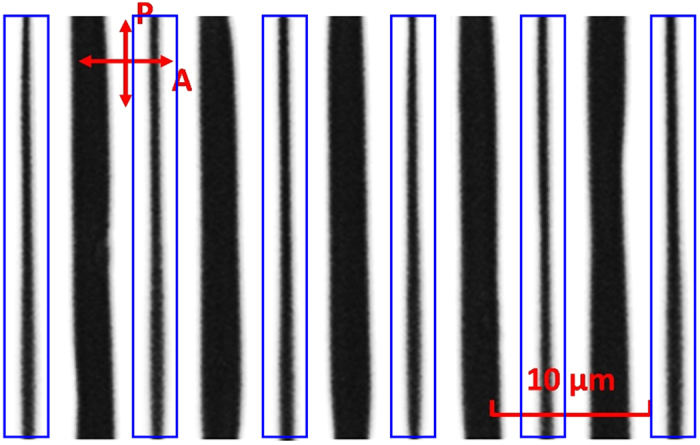
Polarizing optical microscope (POM) image of the fabricated FFS cell under crossed polarizers with an applied voltage *V* = 4.4 V. (P: polarizer; A: Analyzer).

Based on above discussion, no disclination line should occur in our new FFS cell because the LC directors know which direction to rotate. This is confirmed by the experimental results. Figure 7 shows the polarizing optical microscope (POM) image under crossed polarizers with an applied voltage *V* = 4.4 V. As expected, no disclination lines are observed. The dark lines are those stationary regions in the center of electrodes and electrode gaps, due to symmetric LC distributions. To make it more discernable, we also plot the pixel electrode region (blue rectangle) with electrode width *w* = 2.8 μm. In the middle of electrode gaps, there exist wider dark lines, indicating larger stationary regions. This is consistent with the phase profile shown in Fig. 4(a).

## Conclusion

In summary, we have demonstrated a high performance phase grating with switchable diffraction angles using a simple FFS liquid crystal cell. The unique feature of our design is that the rubbing angle is parallel to the FFS electrodes. Such a simple modification leads to ~10X lower operation voltage and ~2X faster response time, as compared to a conventional FFS-based phase grating, in which α ≈ 7°. In contrast to dual-period blue phase gratings, our device offers much simpler driving scheme and >20X lower operation voltage. Potential applications of such a grating for diffraction optics, optical interconnect, and beam steering devices are foreseeable.

## Methods

### Device fabrication

On the bottom glass substrate, transparent interdigitated pixel electrodes and flat common electrodes were formed with an insulating layer (Si_3_N_4_) sandwiched between them. The width and gap for the pixel electrode were 2.8 μm and 6 μm, respectively. The thickness of the insulting layer was ~200 nm. Then a thin polyimide layer was spin-coated onto the inner surface of each substrate and baked at 230 °C for 30 min. Mechanical rubbing process was adopted to get anti-parallel alignment. The rubbing direction was controlled to 0° with respect to the interdigitated electrodes. A small pretilt angle (~2°) was generated by the rubbing process. Then, the silica spacers with a diameter of 3.4 μm were employed to maintain the cell gap uniformly. Finally, an LC mixture with *n*_*o*_ = 1.500, *n*_*e*_ = 1.639, *ε*_*//*_ = 8.0, and *ε*_*⊥*_ = 2.9 was injected into the cell via capillary action.

### Optical characterization

A He-Ne laser (JDS Uniphase, USA) with *λ* = 633 nm was used as probing beam, and a polarizer was placed behind to get linearly polarized light. Its transmission axis was set perpendicular to the pixel electrodes of the FFS cell. An iris was placed behind the FFS cell to select the diffraction order and the intensity of each order was detected by a photodiode (Model 2031, New Focus, USA) in the far-field at a distance of ~30 cm. During the optical measurement, the LC cell was driven by a square-wave voltage at 1 kHz frequency. The applied voltage was controlled by a LabVIEW (National Instruments) system.

## Additional Information

**How to cite this article**: Chen, H. *et al*. A Low Voltage Liquid Crystal Phase Grating with Switchable Diffraction Angles. *Sci. Rep.* **7**, 39923; doi: 10.1038/srep39923 (2017).

**Publisher's note:** Springer Nature remains neutral with regard to jurisdictional claims in published maps and institutional affiliations.
